# Susac syndrome: clinical characteristics, clinical classification, and long-term prognosis: Erratum

**DOI:** 10.1097/MD.0000000000006207

**Published:** 2017-02-17

In the article “Susac syndrome: clinical characteristics, clinical classification, and long-term prognosis”,^[1]^ which appeared in Volume 95, Issue 43 of *Medicine*, Joab Chapman's name was originally misspelled in the byline.
